# Stem cells for organoids

**DOI:** 10.1002/SMMD.20220007

**Published:** 2022-12-27

**Authors:** Shutong Qian, Jiayi Mao, Zhimo Liu, Binfan Zhao, Qiuyu Zhao, Bolun Lu, Liucheng Zhang, Xiyuan Mao, Liying Cheng, Wenguo Cui, Yuguang Zhang, Xiaoming Sun

**Affiliations:** ^1^ Department of Plastic and Reconstructive Surgery Shanghai Ninth People's Hospital Shanghai Jiao Tong University School of Medicine Shanghai China; ^2^ Department of Orthopaedics Shanghai Key Laboratory for Prevention and Treatment of Bone and Joint Diseases Shanghai Institute of Traumatology and Orthopaedics Ruijin Hospital Shanghai Jiao Tong University School of Medicine Shanghai China

**Keywords:** cell surface engineering, gene editing, organoid, stem cell

## Abstract

Organoids are three‐dimensional (3D) cell culture systems that simulate the structures and functions of organs, involving applications in disease modeling, drug screening, and cellular developmental biology. The material matrix in organoids can provide a 3D environment for stem cells to differentiate into different cell types and continuously self‐renew, thereby realizing the in vitro culture of organs, which has received extensive attention in recent years. However, some challenges still exist in organoids, including low maturity, high heterogeneity, and lack of spatiotemporal regulation. Therefore, in this review, we summarized the culturing protocols and various applications of stem cell‐derived organoids and proposed insightful thoughts for engineering stem cells into organoids in view of the current shortcomings, to achieve the further application and clinical translation of stem cells and engineered stem cells in organoid research.

## Graphical abstract

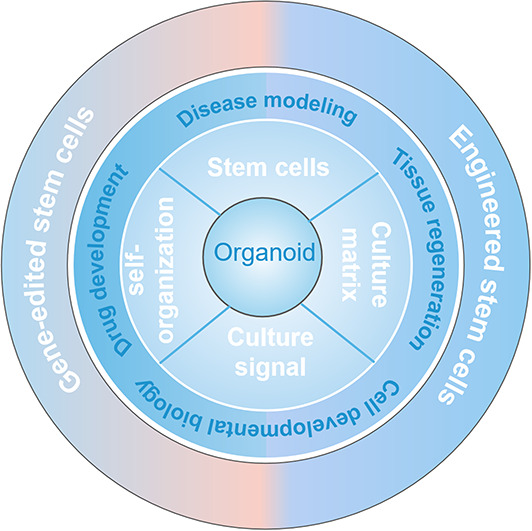

1

**Key points**

- Organoids consist of more than one cell type and can simulate the structure and function of an organ through self‐organization.
- Organoids have been widely applied in disease modeling, cell therapy, tissue regenerration and drug development.
- Various technologies including gene editing, cell‐surface engineering have been used to achieve precise self‐organization of idealized stem cells.
- The purpose of this review was to achieve the further application and clinical translation of stem cells and engineered stem cells in organoid research.

## 2 INTRODUCTION

**FIGURE 1:**
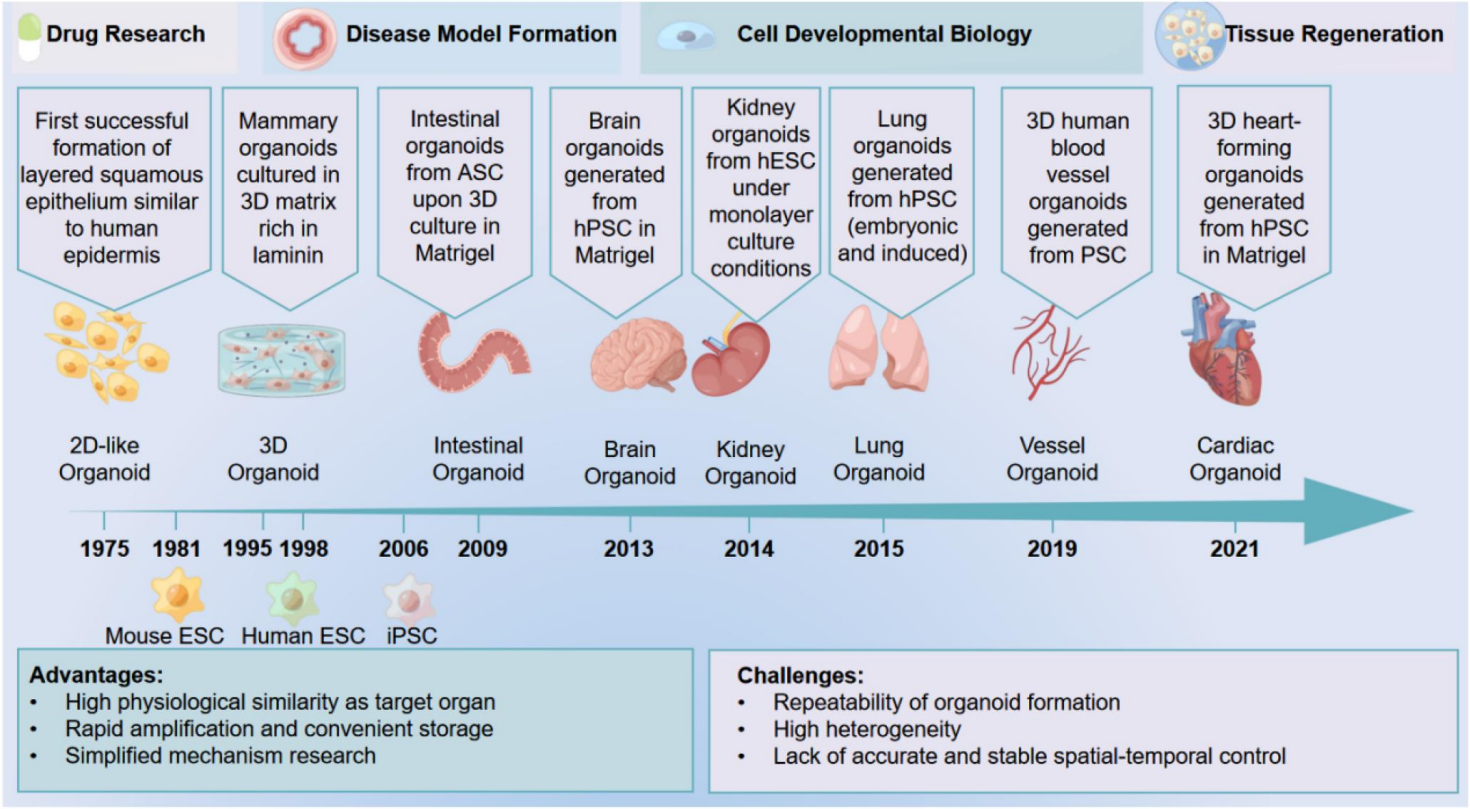
Schematic diagram of the development history of organoids. Organoids have been widely used in disease modeling, drug research, cell development research, and tissue regeneration. Created by FigDraw.

Organoids are complex three‐dimensional (3D) structures developed from somatic cells, adult stem cells (ASCs)/progenitor cells, pluripotent stem cells (PSCs), or specific cell lines that resemble in vivo organs in structures and functions.^1^, ^2^, ^3^, ^4^, ^5^, ^6^, ^7^, ^8^, ^9^, ^10^, ^11^, ^12^, ^13^ In 1946, the term “organoid,” which means “organ‐like,” was first proposed by Smith et al. to describe cystic teratoma.^13^ The first successful construction of organoids originated in 1975, when Rheinwald et al.^1^ pioneered the co‐culture of primary human keratinocytes and 3T3 fibroblasts to form lamellar squamous epithelium similar to human epidermis. However, this cell culture method is much closer to a two‐dimensional (2D) planar culture. The first case of organoids that conformed to the modern concept appeared in the 1990s. It was a mammary organoid cultured in a 3D matrix rich in cohesin constructed by Ronnovjessen et al.^3^ This organoid model held functional alveolar‐like structures with epidermal polarization and directed secretion functions. Currently, organoids are mainly utilized for in vitro organ or disease model construction, drug screening, and regenerative medicine. Since Sato et al.^6^ made a breakthrough in the culture of intestinal organoids^14^, ^15^, ^16^, ^17^, ^18^, ^19^, ^20^, ^21^, ^22^, ^23^, ^24^, ^25^, ^26^, ^27^, ^28^ in 2009, organoids have also been developed in various organs, including the liver,^15^, ^29^, ^30^, ^31^, ^32^, ^33^ kidney,^34^, ^35^, ^36^, ^37^, ^38^, ^39^, ^40^, ^41^, ^42^ lung,^43^, ^44^ heart,^11^, ^45^, ^46^ brain,^47^, ^48^, ^49^, ^50^, ^51^, ^52^, ^53^, ^54^, ^55^, ^56^, ^57^, ^58^, ^59^ ear,^60^, ^61^ retina,^62^, ^63^, ^64^, ^65^, ^66^, ^67^ airway,^68^, ^69^ pancreas,^70^, ^71^, ^72^, ^73^ breast,^3^, ^74^ taste buds,^75^ and ovaries.^76^, ^77^, ^78^ As a significant technological breakthrough, 3D organoid models offer a promising approach to understanding the complexity of human disease from mechanistic studies of disease pathogenesis to treatments (Figure 1).

With the continuous in‐depth exploration of cell developmental morphologies and molecular mechanisms, the construction of in vitro models has gradually become crucial. Traditionally, stem cell differentiation experiments are performed in 2D monolayer culture conditions. Though relatively homogeneous populations of differentiated cells can be generated in 2D culture, it still faces the challenge of low physiological relevance.^79^ This is because the 2D culture systems lack the cell–cell and cell–matrix interactions that require for maintaining and defining in situ phenotypes, thus cannot mimic the cellular functions and signaling pathways present in tissues.^80^ In contrast, 3D cell culture systems have properties that help guide specific functions, growth, and processes of stem cells, such as embryogenesis, morphogenesis, and organogenesis,^81^ so further exploration of 3D culture systems is needed.

Stem cell 3D culture systems can be divided into scaffold‐free culture systems (spheroids) and scaffolded culture systems. Among them, spheroids, as multicellular 3D structures, are mainly applied in research related to cancer stem cells. Spheroids are simple clusters of free‐floating cell aggregates, primarily obtained by culturing under non‐adherent, ultra‐low attachment plates. A remarkable difference between spheroids and organoids is the nature of the driving force during their development. The formation of organoids primarily depends on the internal developmental process, whereas that of spheroids is through cell‐to‐cell adhesion. Besides, spheroids contain a high proportion of poorly differentiated cells, which are analogous with cancer stem cells, making them an excellent model for cancer stem cell research. But spheroids lack the relevant stem or progenitor cell populations demanded to maintain 3D cultures, thus lacking cells with self‐renewal and differentiation capacities.^80^ Organoids are more complex and hold a large number of biological parameters, including cell–cell interactions, cell–matrix interactions, and certain physiological functions produced by tissue‐specific cells in the organoids. In addition, the functions of generated cells are more mature than cells derived from directed differentiation.^5^ Therefore, 3D organoids are helpful for investigating the disease pathologies in the context of spatiotemporal patterns of relevant organism development and can potentially establish drug responses at the organ level rather than the individual cell level, which makes organoid systems more popular in disease model establishment.^13^ In summary, as organoids possess the ability to self‐assemble to form highly heterogeneous and organized structures that can closely mimic morphogenetic processes that occur during in vivo development, they provide an excellent platform for disease modeling, regenerative medicine, cell bio‐development, and pharmaceutical research.

In this review, we will discuss the development of stem cells in organoids construction and give insights into their further improvement, thereby facilitating the future clinical translation of organoids.

## 3 FROM SINGLE CELLS TO ORGANOIDS

Generally, the single cells that compose of organoids include ASCs, embryonic stem cells (ESCs), and induced pluripotent stem cells (iPSCs). The obtained stem cells are usually placed in a 3D culture system, typically a hydrogel (e.g., Matrigel). After supplementing with appropriate exogenous factors, they could mimic the biochemical and physical cues required for tissue development. Eventually, the cells self‐assemble into complicated structures known as organoids. These organoids usually consist of more than one cell type and can simulate the structure of an organ as well as its unique function, this in vitro process is termed as “self‐organization.”

### 3.1 Types of stem cells

The types of stem cell currently used for the construction of organoids include (i) PSCs,^82^, ^83^, ^84^, ^85^ that is, ESCs or iPSCs and (ii) organ‐specific ASCs.^86^ The research on ASCs^27^ focuses on stem cell identification, isolation, expansion in culture via niche factors, and specific lineage differentiation. Unlike ASCs, ESCs or iPSCs can form all body tissues and spontaneously differentiate into a disordered mass of differentiated tissues in vivo.

In 2006, Takahashi et al.^5^ obtained iPSCs from mouse embryonic or adult fibroblasts (ECs) by introducing four transcription factors, Oct3/4, Sox2, c‐Myc, and Klf4. iPSCs are highly like ESCs, both express human pluripotency factors and ESC surface markers and exhibit the developmental potential to differentiate into three germ layers. Compared with ESCs, iPSCs hold several advantages, including easy access, extendibility, ability to generate abundant cell types, avoidance of ethical issues associated with human ESCs, and the potential for personalized medicine.^13^, ^87^, ^88^ However, PSCs also have the limitations that PSC‐derived organoids only occur during embryonic development and are therefore more suitable for studying early organogenesis.

iPSC‐based organoids are established according to the developmental processes, whereas ASCs can form organoids by creating suitable environments that mimic stem cell niches during self‐renewal or repairing of physiological tissues.^89^ Currently, ASC‐derived organoids are mainly applied as a model system for cancer, infection, cystic fibrosis, and other diseases studies.^90^ ASC‐derived organoids are composed of more mature cell types, while iPSCs‐derived organoids maintain an embryonic state. That is why ASC‐derived organoids can hold high genetic stability after long‐term culture. The shortcoming of ASC is that its restricted differentiation potential results in only a single epithelial cell type in all ASC‐derived organoids.^91^

In summary, the culture techniques for generating organoids from PSCs or ASCs are still in their infancy and are highly driven by experience factors. Nowadays, efforts are being made to develop mature organoid systems.

### 3.2 Organoid culture methods

**FIGURE 2:**
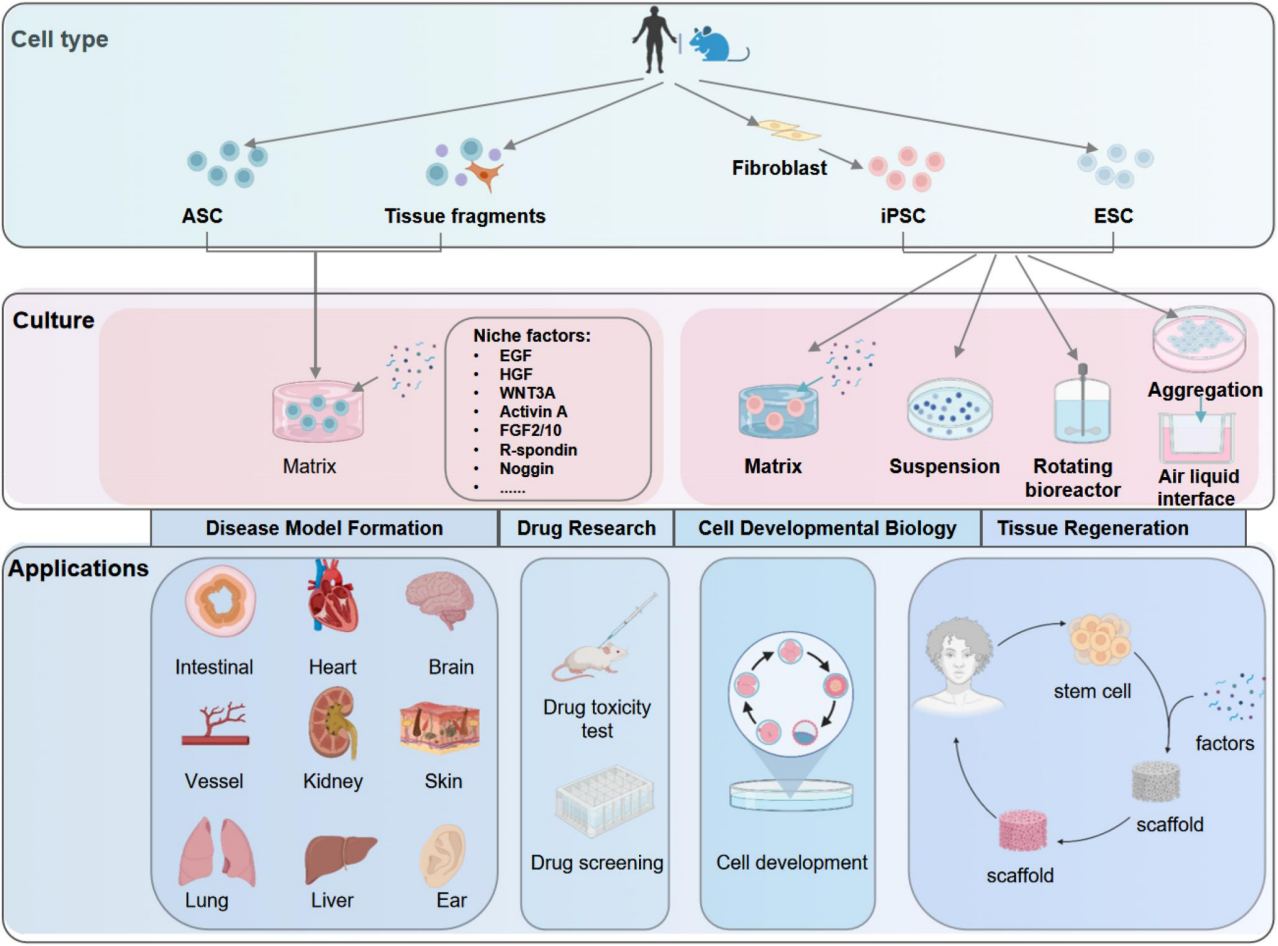
Cultivation procedure, applications, and readout evaluation of organoids. Created by Biorender.

The traditional way of organoid development is to digest tissue stem cells with collagenase, and then culture them in a matrix with medium‐containing growth factors. Others include rotary bioreactors^92^, ^93^, ^94^ and air–liquid interface methods (Figure 2). This section mainly introduces the traditional matrix culture method. In this way, only relevant cell niche factors and a 3D extracellular scaffold are required without other external guidance, stem cells can arrange and generate all progeny of their tissue lineage and self‐organize into 3D structures to mimic the homeostasis of their native organs and functions through a self‐organizing mechanism.^15^, ^90^

#### 3.2.1 Matrix

Matrix is commonly used as a 3D culture environment to serve as the tissue extracellular matrix (ECM), such as the Matrigel, a reconstituted basement membrane extracted from Engelbreth‐Holm‐Swarm (EHS) mouse sarcoma, which plays a critical role in the development of the organoid field. The EHS matrix is a mixture of many different ECM components and other bioactive factors that provide a delicate environment for cell embedding. This matrix has sufficient natural cell adhesion domains to facilitate cell attachment and can be degraded and remodeled by enzymes expressed in the developing organoids. However, EHS matrix varies from batch‐to‐batch production, so it is not suitable for clinical translation and is also not easy for personalized customization.

To avoid possible problems aroused from mouse‐derived matrix hydrogels, researchers turned their attention to other classes of natural^24^, ^95^ or synthetic hydrogels^96^ (Table 1). Glorevski et al.^96^ pioneered utilizing the modular synthetic hydrogel networks to expand the intestinal stem cells (ISCs) derived from mice or humans, which can not only replace animal‐derived gels for organoid culture, but also assist in enhancing the similarity of organoids with real organs. This is the first study of the creative use of synthetic hydrogels for organoid culture.

**TABLE 1:** Applications of natural materials and synthetic polymer materials in different organoids

Matrix type	Material	Cell type	Tissue or organ	References
Natural materials	Collagen	Human pancreatic stellate cells	Pancreas	[98]
		Human iPSC	Intestine	[99, 100]
		Human intestine tissue	Intestine	[101]
	Decellular matrix	Human hepatocarcinoma cells, mesenchymal and endothelial cells	Liver	[102, 103]
		Human iPSC	Pancreas	[104]
	Matrigel	Human iPSC	Blood vessel	[105]
		Human iPSC	Lung	[106]
		Human iPSC	Intestine	[107]
		Human iPSC and endothelial cells	Brain	[108]
		Human ESC	Cardiac	[109]
	Hyaluronic acid	Muring AEC2s	Lung	[110]
	Alginate	Human iPSC	Kidney	[111]
		Human iPSC	Intestine	[112]
	Fibrin laminin	Human iPSC	Alveolar epithelium	[113]
		Murine intestinal epithelial cells	Intestine	[114]
	Silk fibroin	Human iPSC	Kidney	[115]
Synthetic polymer materials	PEG	Human endothelial colony forming cells, MSC	Bone, liver	[116]
		Human hepatocytes and iPSC	Liver	[117]
		Human iPSC and ESC	Intestine	[118]
		Human iPSC	Lung	[119]
	PLA	Adipose‐derived stem cells	Hyaline cartilage	[120]
	PIC	Human liver tissue	Liver	[121]
	PAm	Murine ESC	Cardiac	[122]

Abbreviations: AEC2s, Type 2 alveolar epithelial cells; ESC, embryonic stem cell; iPSC, induced pluripotent stem cells; PAm, polyacrylamide; PIC, polyisocyanopeptides; PLA, polylactic acid.

**FIGURE 3:**
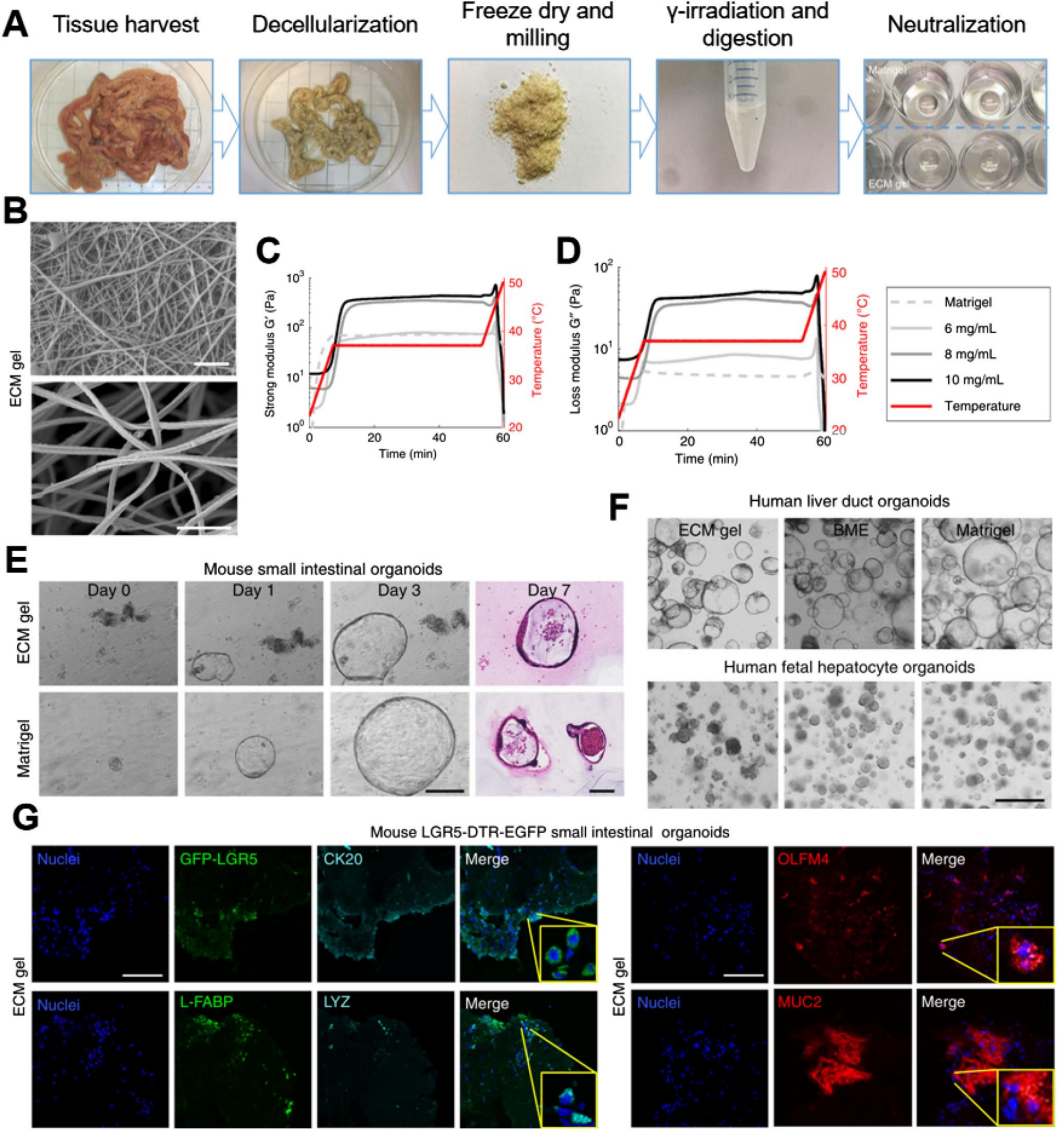
Extracellular matrix hydrogel derived from decellularized tissues.^95^ Reproduced under terms of the CC‐BY license. Copyright 2019, The Authors, published by Springer Nature. (A) The gelation preparation protocol. (B) SEM images of the ECM gel displaying the interconnected fibrous network. Scale bars 1 µm. (C, D) Storage modulus and loss modulus of the ECM gel and Matrigel. (E) Bright field and H&E images of mouse intestinal organoids in ECM gel and Matrigel. Scale bars 100 µm. (F) Culture of liver ductal and hepatocyte human organoids in ECM gel, BME, and Matrigel. Scale bar 500 µm. (G) Immunofluorescence staining of SI organoids in ECM gel after 4 weeks in vivo. Scale bars 100 µm. BME, basement membrane extract; ECM, extracellular matrix; SEM, scanning electron microscope; SI, small intestine.

However, defining all the relevant information needed to guide the remodeling and regeneration of a specific tissue is a real challenge. Still, the synthetic matrix hydrogels can only partially replicate some characteristics of the natural ECM. Therefore, some researchers^95^ used naturally decellularized tissue (DT) materials (Figure 3). As demonstrated by preclinical data and experimental human transplants, cell‐enriched DT could effectively promote tissue regeneration in vivo. Combining organoid technologies with bio‐fabrication techniques would overcome these limitations and generate larger functional tissues under highly controllable culture conditions.

#### 3.2.2 Cell niche signals

During the organoid culturing process, in addition to the matrix, another significant parameter affecting the formation and development of organoids is the cell niche signals that regulate the differentiation of stem cells into specific cell lineages. Since different organs require different niche signals, which cannot be adequately and accurately induced by single incorporation of matrix, several ligands or compounds capable of activating key signaling pathways are often supplemented in the medium to induce the lineage‐specific development of organoids, including epithelial growth factor, bone morphogenetic protein, fibroblast growth factor family, and human hepatocyte growth factor,^97^ etc. (Figure 1).

#### 3.2.3 Symmetric breaking and self‐organization

As for stem cells, symmetry breaking must occur to self‐organize from simple spherical cells to form organoids with complicated tissue structures. For instance, intestinal organoids^123^ are developed from LGR5+ stem cells. First, stem cells generate symmetrical spherical structures, then Paneth cells emerge to create a niche and secrete WNT3A. After the broken symmetry event, only a tiny fraction of identical cells in the symmetrical spheres differentiate into Paneth cells, generating a stem cell niche with a WNT3A gradient around the Paneth cells, which in turn induces the formation of crypt and villus structures. This process is the first and most critical symmetric broken event during the construction of intestinal organoids. Generally, it involves some degree of random differentiation, cell sorting, and feedback between adjacent cells to establish boundaries and signaling centers, a self‐organized process in which cells with similar adhesion properties tend to associate with each other.^124^ This theory is termed the differential adhesion hypothesis by Lancaster.^125^

Overall, PSCs or ASCs can gradually form organoids from single cells through the 3D environmental support of the matrix, the modulation of niche signaling, and the self‐organization mechanisms of symmetry breaking. These components also provide crucial clues for the further upgrading of organoids.

## 4 APPLICATION OF ORGANOIDS

As 3D cell culture systems that simulate organ structures and functions, organoids involve various applications, such as disease modeling, cell therapy, and drug screening.

### 4.1 Disease modeling of organoids

After the successful construction of intestinal organoids, more and more different organoids have been established and applied to the modeling of genetic and infectious diseases. Menendez et al.^126^ constructed kidney organoids from a patient with polycystic kidney disease, laying the foundation for the study of other hereditary renal diseases. Similarly, Hofbauer et al.^11^ established hPSC‐derived NKX2‐5 and HAND1 knockout cardiac organoids mimicking the hypoplastic left heart syndrome. Organoids related to other genetic disorders such as Alzheimer's disease, Parkinson's disease, and microcephaly have been successfully established. In the future, organoids will provide more convenient research approaches for disease modeling. In addition, researchers can further explore genetic defect‐related diseases by combining with gene‐editing technologies such as CRISPR/Cas9.

### 4.2 Drug development of organoids

In drug screening, 2D culture systems lack tissue structures and complexity, while organoids can highly recapitulate disorder‐derived features with higher sensitivity, heterogeneity, and stability. Furthermore, organoids can be preserved, renewed, delivered indefinitely, and mechanically cultured on a chip for drug screening and are promising platforms for development of new drug candidates.^12^, ^127^ Using microfluidic chips, intestinal organoids and human gut microbiota were co‐cultured by Jalili‐Firoozinezhad et al.^127^ Physiologically relevant oxygen gradients were assessed in real time. The results showed that the luminal hypoxia gradient established in the organoid chip increased intestinal barrier function and maintained a physiologically relevant level of microbial diversity. Therefore, intestinal organoids could serve as a tool for the development of microbiome‐associated therapies, probiotics, and nutraceuticals. In addition, certain limitations still exist in the toxicity evaluation and preclinical research of drugs at this stage, leading to the gradual discovery of toxic effects of many medicines in clinical research or marketing stage. Organoids can be utilized to build a long‐term toxicity screening model with the characteristics of human physiology to estimate drug toxicity because of their similar responses to those of physiological tissues.

In conclusion, organoid technologies provide a valuable tool for potential high‐throughput drug screening, enabling accurate toxicity evaluation and preclinical studies.

### 4.3 Tissue regeneration of organoids

The limitations of donor sources for organ transplantation have sparked research into the development of functional alternatives to organs and the breakdown of organoids could advance the development of regenerative medicine. For tissue regeneration, obtaining mature organoids is critical to achieve vascularization, reduce unnecessary differentiation after transplantation, and promote functional reconstitution.^81^ Intestinal organoids are a relatively mature system among organoid models, which were first applied in regenerative medicine. For example, Cruz‐Acuna et al.^128^ encapsulated hPSCs into a PEG hydrogel matrix, which was set as the carrier, to yield intestinal organoids. Then, they injected this hPSCs‐loaded hydrogel into the damaged intestinal mucosa for significantly improved closure of colonic mucosal wounds. However, the current organoid derivation‐related strategies mainly focus on the production of microscale organoids. At the macroscale organoids (i.e., 100 μm to millimeters), there are obstacles in various aspects, including their morphology, the difference between organoids and real organs in cellular structure and composition, and poor reproducibility. Therefore, it is in demand to be combined with cell engineering, biofabrication, and other technologies in the future to truly realize the mature translation in the field of tissue regeneration.

### 4.4 Cell developmental biology of organoids

During embryogenesis, the developmental process strongly depends on complicated networks and communications of cell–cell and cell–matrix interactions between different cell populations and their microenvironments. These cross talks affect cellular behaviors such as proliferation, survival, migration, and differentiation; therefore, the development of organoids paves the way for investigating the developmental biology. Ng et al.^129^ used inverted colloidal crystals (ICCs) to engineer a liver organoid platform of which 3D mechanical properties were fabricated to recapitulate the extracellular niche surrounding hepatic progenitor cells during human development. Leibel et al.^130^ found out the embryonic developmental cues by introducing multiple growth factors and small molecules to efficiently generate final endoderm, foregut pre‐endoderm, and subsequent lung progenitors. Overall, considering the excellent abilities of organoids to construct critical features of real organs, they have become the powerful model systems for studying organ development at the cellular level.

## 5 ADVANTAGES AND DISADVANTAGES OF ORGANOIDS

Regarding the advantages of organoids, first of all, unlike traditional in vitro culturing methods, organoids are similar in composition and structure to primary tissues, containing a small number of genomically stable and self‐renewing stem cells, of which the progenies are similar to these of living tissues.^30^ Second, organoids can be rapidly expanded, cryopreserved, and applied in the high‐throughput analysis. Furthermore, organoids are an important bridge between traditional 2D cultures and in vivo mice models since they are more physiologically relevant than monolayer cells and easier to manipulate niche components, signaling pathways, and gene editing than in vivo models.^131^

The shortcomings of organoids include the following: (1) Low reproducibility. Subramanian et al.^132^ analyzed and compared four different organoids using the single‐cell RNA sequencing method, demonstrating significant differences among different iPSC lines, protocols, and experimental batches. The lower reproducibility would restrict the application of organoids in the studies of drug screening or cell molecular mechanisms.^133^ (2) High heterogeneity. Due to the lack of specificity of cell types and the high dependence of organoid cells on their “self‐organization,” a lengthy and precise procedure is required to produce suitable organoid‐derived cells, which results in the high heterogeneity of cells and organoids. (3) The lack of spatiotemporal regulation during the development of the organoids^30^ could limit the size of organoids to the centimeter level, which in turn restricts their clinical application.

## 6 STEM CELLS REMODELING

The emergence of organoids provides an excellent platform for disease modeling, regenerative medicine, cell biology, and drug research. However, it can still not obtain tissues or organs that are perfectly similar to those in the physiological state by in vitro culture. Ideally, without extra external guidance, but only by the assistance of appropriate niche factors and 3D scaffolds, stem cells can generate all progeny of their tissue lineage and self‐organize into 3D structures to mimic the homeostasis and functions of their native organs. However, either PSC or ESC cannot achieve accurate and stable tissue self‐organization by solely relying on stem cell self‐organization, which leads to the high heterogeneity of organoids. Many researchers make an effort in overcoming the challenge of high heterogeneity by modulating 3D environments and niche signaling, of which the most important is to modify the matrix, such as 3D bio‐printing, hydrogels, and microfluidics. However, the precise self‐organization of idealized stem cells cannot be achieved simply by the current biomaterials. Therefore, it may potentially focus on how to engineer the surface of stem cells or carry genes to realize high precision.

### 6.1 Engineered stem cells

**FIGURE 4:**
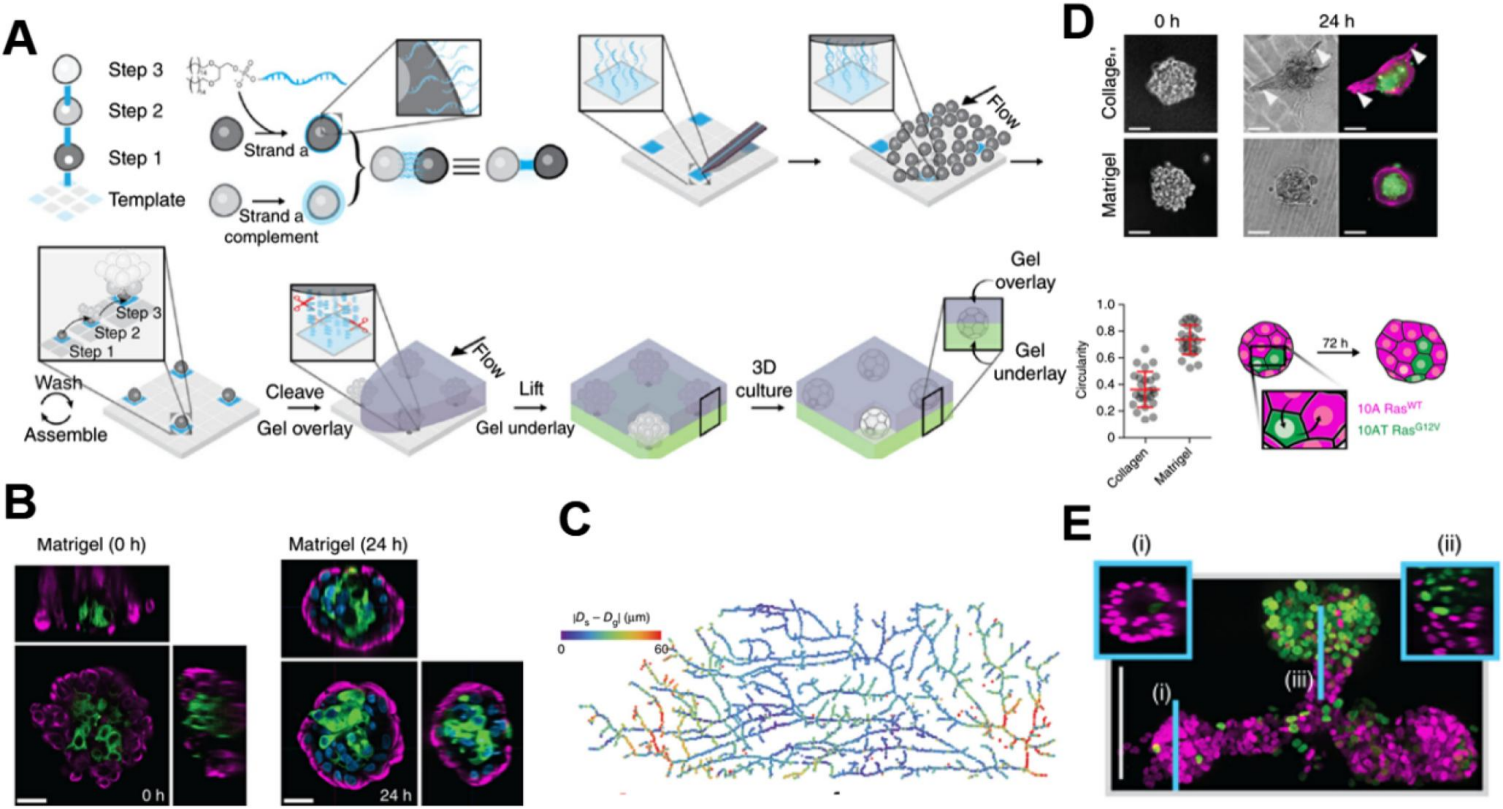
Programing the reconstitution of fully ECM‐embedded 3D microtissue arrays by DNA‐programed assembly (DPAC).^134^ Reproduced with permission. Copyright 2015, Springer Nature. (A) Scheme showing the relationship between DNA spots (colored squares), DNA‐programed connectivity (colored lines), and multistep assembly. (B) Images of fully embedded aggregates of human luminal and myoepithelial cells. (C) Heat map illustrating differences in global cell position in two dimensions versus three dimensions relative to the pattern center. (D) Representative images of human mammary luminal and myoepithelial cells assembled through identical four‐step synthetic schemes and then transferred to Matrigel or collagen I. (E) Maximum‐intensity projection of a center‐patterned microtissue after processing using CLARITY. Insets are single confocal sections of the indicated region of the microtissue. ECM, extracellular matrix.

Over the past few decades, efforts have been devoted to developing biomaterials that can regulate the behavior of stem cells, thereby mimicking or reorganizing the ECM in various ways. With the concept of the biological interface, control of stem cell fate can be achieved through cell surface engineering to enhance cell adhesion and direct differentiation. For example, Todhunter et al.^134^ used lipid‐modified oligonucleotides to bind amine groups on the cell surface to precisely realize DNA surface mapping. Then, DNase‐containing Matrigel was added to cleave the DNA, and the microtissue arrays were released into the ECM. This offers an opportunity to achieve the precise spatial arrangement of a large number of single cells and whole microtissues (Figure 4).

Chemical modification on the cell surface or layer‐by‐layer self‐assembly through electrostatic interaction forces could promote the self‐organizing ability of stem cells at the microscale level, and then construct organoids that resemble tissues or organs in a physiological condition. These engineered stem cells prepared by surface engineering strategies show a promising prospect for organoids construction in the future.

### 6.2 Gene‐edited stem cells

The genetic engineering techniques allow unprecedented regulation over organoid development via perfect control of gene expression and mutation. There are two common methods to transfer genes into organoids: viral (such as retroviral/lentiviral or adenoviral transduction) and naked DNA transfer nonviral (electroporation/lipolysis). Adenovirus or electroporation can be used when transient gene expression needs to be changed. When permanent changes are needed, lentivirus or transposon can be applied for gene transfer or CRISPR/cas9 system can be used for gene editing. For example, Liu et al.^135^ developed a model of retinoblastoma in retinal organoids that were derived from engineered human embryonic stem cells (hESC) with biallelic mutation of the RB1 gene. The results showed that the characteristics of organoids are highly consistent with the occurrence, transcriptome, and genome‐wide methylation of retinoblastoma.

**FIGURE 5:**
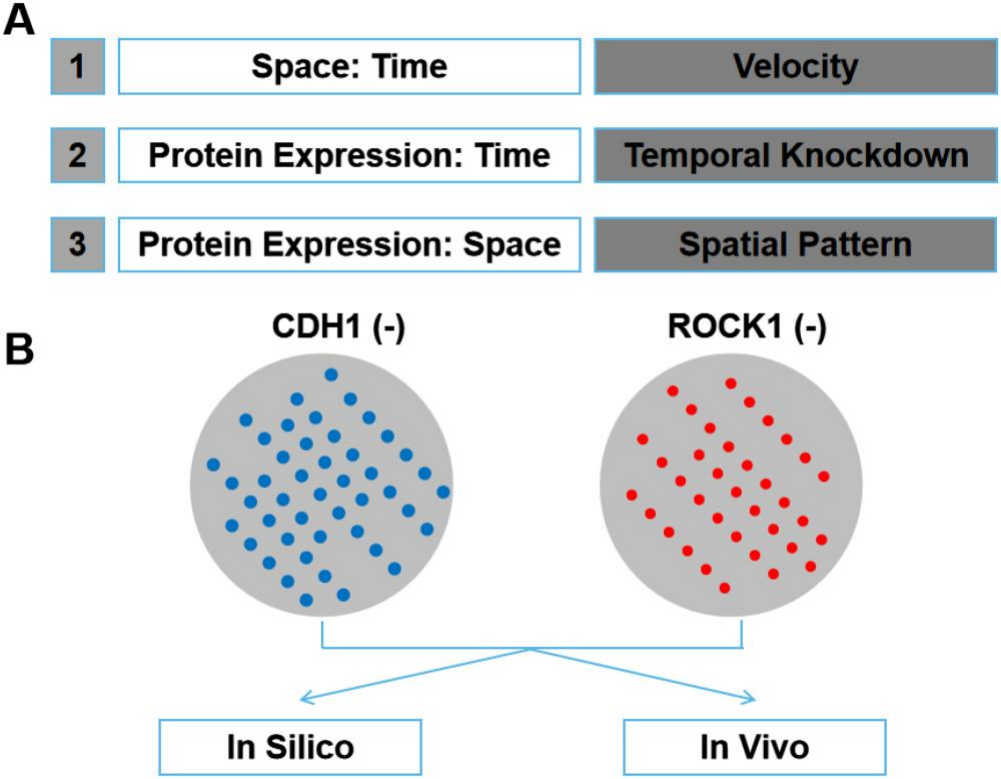
A computational model of PSC dynamics, enabling a machine learning optimization approach to predict experimental conditions. (A) Space–time relationships are captured with velocity characterizations, time–protein expression is captured characterizing the relative protein expression for several days after knockdown, and protein–space relationships are characterized by confocal microscopy imaging of spatial behavior due to cell mechanical perturbations. (B) Paired in vitro and in silico, schematic images of spatial patterning after CDH1 (blue) and ROCK1 (red) knockdown in stem cells.^135^ Reproduced with permission. Copyright 2020, The Authors, published by the National Academy of Sciences of the United States of America PSC, pluripotent stem cells.

It is reported that^136^ CRISPR interference (CRISPRi) technology was used to knock out genes related to organoid morphology at different stages of cell cluster evolution. Extensive experiments are required considering the potential time points at which all genes may be knocked out, the proportion of targeted cells, and other variables. Therefore, this research utilized the artificial intelligence technique to build a machine learning model that can quickly acquire the optimal gene knockout groups and protocols (Figure 5). In this work, active multicellular tissue can be induced by controlling the disturbance of internal cellular mechanisms without modifying the matrix, which means that in the future, with the help of CRISPR technology, machine learning and mathematical optimization, researchers can predict and control the spatial self‐organization of heterogeneous populations of pluripotent cells with a more accurate method.

## 7 CONCLUSION

The 3D environment of organoids contains a large number of biological processes, including cell–cell interaction, cell–matrix interaction, and some physiological functions produced by tissue‐specific cells in organoids. Due to its unique 3D micro‐environment and self‐assembly ability, organoids can more accurately simulate the internal environment. In particular, organoids are similar in composition and structure to primary tissues, including a small number of genetically stable and self‐renewing stem cells, of which the genes are similar to those of living tissues. It can be said that the emergence of organoids provides a good platform for disease modeling, regenerative medicine, cell biological development, and drug research. However, at present, it is impossible to obtain tissues or organs that are completely similar to the physiological state through in vitro culture, mainly due to the low self‐assembly ability of stem cells. Ideally, through the arrangement of the self‐organization mechanism, there is no need for external guidance, but only the provision of appropriate niche factors and a 3D extracellular scaffold. Stem cells can produce all descendants of their tissue lineage and self‐organize into a 3D structure to imitate the stability and function of their primary organs. Unfortunately, relying solely on cell self‐organization cannot achieve accurate and stable tissue self‐assembly, whether PSC or ESC, which leads to high heterogeneity of organoids.

In most cases, researchers choose to compensate for heterogeneity through the regulation of a 3D environment and niche signals. In this review, we shifted attention to the research on stem cell remodeling. In the past decades, people have been committed to developing biomaterials that can control cell behavior, so as to simulate or recombine ECM in various ways. With the concept of biological interface proposed, the fate of cells can be controlled by cell surface engineering to enhance cell adhesion and direct cell differentiation. Cell surface engineering can realize the precise spatial arrangement of a large number of single cells and the whole microstructure on the microscale through chemical modification or electrostatic interaction on the cell surface.

Meanwhile, genetic engineering technology can control organoid development unprecedentedly by accurately controlling gene expression and mutation. Through the gene editing technique, the gene related to organ shape or function could be knocked out. Besides, with the combination of artificial intelligence, the better gene knockout group and plan could be quickly developed. Although it was still difficult to construct the organs above the centimeter level, with the progress of the cell remodeling technique, it could be predicted that the organs would develop rapidly.

## AUTHOR CONTRIBUTIONS

Shutong Qian and Jiayi Mao are responsible for main literature collection and review writing. Zhimo Liu, Binfan Zhao, Qiuyu Zhao and Bolun Lu are responsible for part of literature collection. Liucheng Zhang and Xiyuan Mao are responsible for review and modification of the manuscript and funding acquisition. Liying Cheng is responsible for supervision. Wenguo Cui, Yuguang Zhang and Xiaoming Sun are responsible for supervision and funding acquisition.

## CONFLICT OF INTEREST

The authors declare that they have no conflict of interest. Wenguo Cui is a member of the *Smart Medicine* editorial board.
